# Clinical study on gray matter volume reduction, gait disorders, and fall risk in patients with Alzheimer’s disease

**DOI:** 10.3389/fneur.2026.1737591

**Published:** 2026-02-19

**Authors:** Shan Wang, Yihan Yang, Kexin Cheng, Xiaotong Yang, Xuelin Wang, Ya Liu, Yi Qiao, Wenjie Gao, Ya Wen

**Affiliations:** 1 Department of Neurology, The Second Hospital of Hebei Medical University, Shijiazhuang, Hebei, China; 2 Key Laboratory of Clinical Neurology, Ministry of Education, Hebei Medical University, Shijiazhuang, Hebei, China; 3 Neurological Laboratory of Hebei Province, Shijiazhuang, Hebei, China; 4 Department of Critical Care Rehabilitation, Hebei Provincial People’s Hospital, Shijiazhuang, Hebei, China

**Keywords:** Alzheimer’s disease, fall, gait, gray matter volume, risk factors

## Abstract

**Objective:**

Falls represent a major threat to the physical and mental health of older adults. This study is dedicated to determining the relationships of fall risk with reduced gray matter volume and gait disorders in patients with amnestic mild cognitive impairment (aMCI) and Alzheimer’s disease (AD).

**Methods:**

24 aMCI and 21 AD patients were recruited from the Neurology Department of the Second Hospital of Hebei Medical University from January 2024 to December 2024. A prospective nested case–control design was employed on eligible participants for general data collection, neuropsychological testing, gait analysis [including single-task walking (STW) and dual-task walking (DTW)], structural MRI and following up for 6–12 months. The primary outcome was fall that occurred during the follow-up period (recorded through telephone follow-up), and the secondary outcomes were differences in baseline cognitive function, gait parameters, and brain structure between the fall group and the non-fall group. Subjects were categorized as fallers or non-fallers based on incident falls during follow-up. Univariate analysis was performed to screen for potential risk factors contributing to falls. Logistic regression analysis was employed to identify the independent risk factors for falls in aMCI and AD. Furthermore, disparities in gray matter volume between the fall and non-fall groups were investigated by voxel-based morphometry (VBM) analysis (the false discovery rate (FDR) correction based on clumps was adopted, *p* < 0.05).

**Results:**

In this preliminary queue with limited sample size, body mass index (BMI), stride length variability, and medication burden were found to be independent predictors of incident falls by univariate and multivariate analyses. The results of VBM suggested significantly reduced gray matter volume in fallers within the left cerebellar Crus I, lobule VI, and fusiform gyrus; right cerebellar lobule VI, fusiform gyrus, and lobule IV–V; and right superior and middle temporal gyrus, compared with non-fallers.

**Conclusion:**

The preliminary findings of this study indicate that BMI, stride length variability, and medication burden may be associated with fall risk and reduced gray matter volume in specific brain region may be a potential neuroanatomical basis for increased risk of falls in AD patients, which need to be further validated in larger cohorts.

## 1 Introduction

With the accelerating global aging trend, healthcare priorities for the elderly have shifted from single-disease management to comprehensive interventions addressing multisystem comorbidities. In this context, falls, as a critical manifestation of geriatric syndromes, have progressively become a hot topic in terms of their underlying mechanisms and preventive strategies (1). Although falls are prevalent among the elderly population, patients with neurodegenerative diseases face compounded risk due to their unique pathophysiologic features (2). Alzheimer’s disease (AD), a common age-associated neurodegenerative disease, is classically defined by progressive cognitive decline. However, owing to shared neuroanatomical substrates for cognitive and motor functions, a substantial proportion of patients exhibit motor abnormalities, including reduced gait velocity and increased gait variability, which can culminate in falls (3). Recent, falls are increasingly recognized as events reflecting cognitive-motor interactive imbalance, and the risk factors leading to falls of AD patients have not been explicated.

Existing studies indicate that older adults with dementia experience falls at eight times the rate of cognitively intact peers, with significantly higher incidence and severity of fall-related complication (4). Multiple fall risk assessment scales have been developed, including the widely utilized Morse Fall Scale and Fall Risk Assessment for Older Adults; however, their sensitivity in cognitively impaired populations requires further validation. By contrast, international research has focused more extensively on sensory function, balance status, and gait characteristics, and other factors. Gait impairment, one of the determinants of motor dysfunction, is ubiquitous in dementia progression and parallels cognitive decline. Oliveira Silva et al. suggested that the Timed Up and Go (TUG) test could serve as a screening tool for fall risk by quantifying gait performance in individuals with cognitive impairment (5). Nevertheless, while TUG is a standard screening instrument, its predictive capacity for community-dwelling older adults is limited, and it should not be used in isolation (6). Zhang et al. (7) reported that gait variability during dual-task conditions predicted future falls in community-dwelling older adults over a 2-year follow-up, whereas gait velocity did not. Sheridan and Hausdorff (8) further established that stride time variability partially explains the propensity for falls in AD patients. Accumulating evidence supports gait parameters-particularly gait variability, which exhibits superior predictive value over conventional metrics such as gait velocity-are valuable screening tools for identifying high-risk fall individuals suffering AD.

At present, the potential mechanisms underlying fall risk in cognitive impairment remain incompletely elucidated. There is contradictory evidence of the association between cognitive impairment and falls. It was reported that memory deficit was associated with increased fall risk in the elderly with aMCI and mild AD (9). It was also reported that cognition was not associated with the fall outcome in older adults (10). Our study preliminarily compared the cognitive domain differences between falls and non falls in AD through detailed neuropsychological assessment. Moreover, altered brain structure may contribute to explain the risk of falls in AD patients. Studies have demonstrated that AD fallers exhibited more severe periventricular white matter hyperintensity (11) and smaller hippocampal volumes (12) compared with non-fallers. Furthermore, several studies have demonstrated that reduced volumes in multiple brain regions correlated with heightened fall risk in MCI and AD patients (9, 13).

Strengths of this study include the prospective ascertainment of incident falls over 6–12 months, strengthening causal inference regarding predictors, and the novel application of VBM to identify structural brain alterations associated with falls in individuals with aMCI and AD, providing new insights into potential neural mechanisms. Besides, to clarify the relationship between cognitive impairment and fall risk, our study preliminarily compared the cognitive domain differences between falls and non falls in AD. In summary, our study preliminarily investigated fall risk factors in a limited sample AD queue through integrating neuropsychological assessments, quantitative gait analysis with structural neuroimaging data. We aimed to investigate whether gait parameters (particularly stride length variability) were the independent predictors of falls and validate the hypothesis that reduced gray matter volume in specific brain region may be associated with increased risk of falls in AD patients.

## 2 Materials and methods

### 2.1 Study participants

Participants with aMCI and AD were recruited from the Neurology Department of the second hospital of Hebei Medical University from January 2024 to December 2024 and approved by Research Ethics Committee of the second hospital of Hebei Medical University (ethical approval codes 2024-C029-F1 and date January 1, 2024). Most resided in the urban area of Shijiazhuang. Inclusion criteria: (1) aMCI: Diagnosis according to the National Institute on Aging and the Alzheimer’s Association (NIA-AA) criteria for MCI due to AD (2011) (14). (2) AD: Diagnosis according to the NIA-AA criteria for AD (2011) (15). Exclusion criteria: (1) history of conditions impairing balance (e.g., central neurological disorders, neuromuscular diseases, significant ocular pathologies); (2) other conditions potentially causing cognitive decline (e.g., other types of dementia, chronic heavy alcohol use, severe anemia, neurosyphilis, folate or vitamin B12 deficiency, neurodevelopmental disorders); (3) musculoskeletal or recent traumatic conditions significantly affecting gait (e.g., severe osteoarthritis, inflammatory arthritis, recent fractures); (4) history of cerebral infarction or cerebral hemorrhage with causative lesions confirmed on 3.0T MRI; (5) contraindications to MRI scanning (e.g., metallic implants such as cardiac stents or orthopedic hardware); (6) Inability to comply with study protocols (e.g., due to paralysis, schizophrenia, or severe aphasia). The total number of individuals screened was 56 cases. Five cases were excluded: one case failed to complete magnetic resonance imaging because of metal dentures, two cases were unable to complete dual task gait analysis and one was illiterate. Five cases did not agree to participate in our study. One case dropped out before completing follow-up.

### 2.2 Study methods

In this study, a prospective nested case–control design was employed on eligible participants for general data collection, neuropsychological testing, gait analysis [including single-task walking (STW) and dual-task walking (DTW)] and structural MRI. Patients with aMCI and AD were followed for 6–12 months, and fall events were recorded via telephone follow-up, and participants were categorized as fallers or non-fallers based on incident falls during follow-up. For each faller during follow-up, non-fallers from the same cohort were matched to each faller by sex, age (±5 years), and Mini-Mental State Examination (MMSE) score (±3 points) in a 1:2 ratio to form the control group. Group comparisons examined differences in cognitive domains, gait parameters, and structural MRI measures. We also explored variables that could independently predict future falls.

#### 2.2.1 General information collection

Comprehensive baseline data were collected, including: age, sex, education level (years), body mass index [BMI; calculated as weight (kg)/height (m)^2^], medication burden, and history of falls in the preceding year. According to the Chinese adult BMI standard, body weight is divided into four categories (underweight <18.5; normal 18.5 ~ 23.9; overweight 24.0 ~ 27.9; obesity ≥28.0).

Medication burden was defined as the total number of prescribed medications within the following classes: AD-specific cognitive treatments [cholinesterase inhibitors (ChEIs), NMDA receptor antagonists], antidepressants, antipsychotics, and benzodiazepines.

#### 2.2.2 Neuropsychological assessment

Neuropsychological scale assessments were administered by a board-certified neurologist trained in standardized protocols, evaluating both global cognition and specific cognitive domains.

(1) MMSE: the Chinese version of the MMSE (Zhang Mingyuan-revised version) was administered. This version contains the following components: Orientation (10 points), Immediate Recall (3 points), Calculation (5 points), Delayed Recall (3 points), Language (8 points), and Structural Imitation Task (1 points), with a total score of 30 points.
(2) World Health Organization-California Verbal Learning Test (WHO-CVLT): during the administration protocol, examiners orally presented a 15-word list in identical order across four consecutive learning trials. Immediately following each presentation, patients orally recalled as many words as possible, with examiners recording both the quantity and accuracy of responses. This study implemented three immediate recall trials, followed by a mandatory 15-min delay interval. After this delay, patients again attempt to recall the target words, with correct responses documented verbatim. The primary outcome measure analyzed in this study was the composite CVLT score, calculated as the sum of words recalled across all three learning trials plus the delayed recall trial score. Clinically, this instrument specifically evaluates verbal episodic memory function, assessing encoding, retention, and retrieval mechanisms through structured list-learning paradigms.
(3) Digit Span Test (DST): this test comprises forward and backward span subtests. During administration, the examiner orally presents digit sequences beginning with 3-digit combinations and progressively advancing to 12-digit combinations. Each sequence length includes two trials. The patient must accurately repeat digits in presented order for forward span. If the first trial at a given length fails, a second trial is administered. Testing was discontinued after two consecutive failed trials at any sequence length or upon successful completion of the maximum 12-digit span. The backward subtest follows identical procedures except requiring reverse-order repetition. Scoring reflected the longest sequence length correctly recalled for each subtest. For analysis, we used the sum of the raw scores from both the forward and backward subtests. The DST evaluates working memory capacity, attentional control, and executive function.
(4) Trail Making Test (TMT): this test comprises two parts. Part A requires patients to sequentially connect numbered 1 to 25 in ascending order within 150 s. Part B requires alternating connections between white circles enclosed in black circles (e.g., white circle 1 → black circle 1 → white circle 2 → black circle 2) within 300 s. For both conditions, completion time (seconds) and accuracy are recorded. This study analyzed the derived metric TMT B-A time (Part B completion time minus Part A time). TMT A primarily assesses processing speed and visual scanning, while Part B additionally evaluates task-switching and executive control. The B-A difference score is thought to better isolate executive function components from basic processing speed (16).
(5) Boston Naming Test (BNT): the Chinese adaptation, comprising 30 pictorial items presented sequentially, was administered. Patients verbally name each image spontaneously, with the number of correct responses recorded without semantic or phonemic cues. This instrument specifically evaluates the patient’s language function.

Raw scores from all tests were analyzed. Note that a higher TMT B-A time score reflects a larger time difference, indicating poorer executive function. Conversely, higher scores on the MMSE, CVLT, DST, and BNT indicate better performance in their respective domains.

#### 2.2.3 Gait parameter assessment

Participants walked at their self-selected pace along a well-lit, unobstructed walkway. Each trial involved walking 3 m from the start to an endpoint marker, executing a 180° turn, and returning to the start point (total 6 m per lap). Participants performed three consecutive uninterrupted laps, resulting in a total walking distance of 18 m. Gait parameters were assessed under three conditions: (1) single-task walking (STW): walking only; (2) dual-task walking-calculation (DTW-Cal): walking while serially subtracting 3 from 100; (3) dual-task walking-verbal fluency (DTW-VF): walking while verbally generating names of animals. Participants were instructed to prioritize continuous walking during dual-task trials, even if it meant pausing or slowing down the cognitive task. Prior to formal data acquisition, all participants completed supervised practice trials under researcher guidance. Throughout testing, researchers accompanied participants to ensure safety. Participants were instructed to perform both walking and cognitive tasks to their maximum ability simultaneously. Participants were not permitted to use any assistive devices during walking. Gait data were reviewed by a trained technician following test completion to ensure data collection accuracy.

Gait assessment was performed using the ReadyGo™ Quantitative Motor Function System (hereafter referred to as ReadyGo), which utilizes ultra-high-resolution depth-sensing cameras coupled with artificial intelligence algorithms for motion analysis. It tracks full-body skeletal points in real-time, quantifying kinematic parameters and automatically identifying key gait events (e.g., foot strike, foot-off, turns). The system computes and outputs gait metrics including stride velocity (cm/s), step height, step width, and stride length variability. Furthermore, stride velocity dual-task cost (DTC) was calculated using the standardized formula: stride velocity DTC (%) = [(Stride Velocity _STW_ − Stride Velocity _DTW_)/Stride Velocity _STW_] × 100.

#### 2.2.4 Magnetic resonance imaging acquisition and preprocessing

Whole-brain MRI data were acquired using a Philips Achieva 3.0T scanner equipped with an 8-channel phased-array head coil. High-resolution 3D T1-weighted structural images were acquired with the following parameters: repetition time = 6.2 ms; echo time = 2.4 ms; inversion time = 1,000 ms; flip angle = 8°; field of view = 256 × 256 mm^2^; matrix = 256 × 256; slice thickness = 1 mm; isotropic voxels = 1 × 1 × 1 mm^3^; 160 sagittal slices; acquisition time = 5 min 41 s.

Voxel-based morphometry (VBM) preprocessing was performed using the CAT12 within SPM12 running on MATLAB R2022a. Data underwent quality control followed by format conversion with origin adjustment. Segmentation of acquired high-resolution 3D-T1 images included: (1) format conversion: Digital Imaging and Communications in Medicine (DICOM) to NIfTI; (2) segmentation of T1 anatomical images into gray matter (GM) and white matter (WM) in native space using tissue probability maps (TPMs); (3) image segmentation: removal of non-brain tissues followed by brain tissue segmentation into cerebral GM, WM, and cerebrospinal fluid (CSF) based on standard GM/WM templates; (4) nonlinear correction: nonlinear normalization preserving absolute intracranial volume to generate adjusted GM and WM images; (5) spatial smoothing: the gray matter was smoothed by using 8 mm half-height and full-width Gaussian kernel; after preprocessing, between-group statistical analyses were conducted; (6) results were visualized using the XJVIEW toolbox.

Total Intracranial Volume (TIV; cm^3^) was estimated from the native-space segmented images using the CAT12 software pipeline.

#### 2.2.5 Fall follow-up

Participants were prospectively followed for 6–12 months after the baseline assessment. Fall incidents were ascertained during monthly telephone follow-up interviews using a standardized questionnaire. A fall was defined according to the World Health Organization (WHO) as “an unexpected, unintentional change in position resulting in landing on the ground, floor, or other lower level” (1). Participants reporting one or more falls during the follow-up period were classified as fallers. Details of each fall event were recorded. Falls attributed to acute medical events (e.g., palpitations, sweating, dizziness, debilitation) were excluded from analysis.

#### 2.2.6 Statistical analysis

Statistical analysis was conducted in SPSS statistical software. Participants were categorized as fallers or non-fallers based on incident falls during follow-up. Normality of continuous variables was assessed using the Shapiro–Wilk test and visual inspection of histograms. Normally distributed data are presented as mean ± standard deviation (SD) and compared between groups using independent samples *t*-tests. Non-normally distributed data are presented as median [interquartile range (IQR)] and compared using Mann–Whitney *U* tests. Categorical variables are presented as frequencies (percentages) and compared using Pearson’s *χ*^2^ test or Fisher’s exact test, as appropriate. The significance threshold was set at *p* < 0.05. Variables showing a significant association with faller status (*p* < 0.05) in univariate analyses or deemed clinically relevant were entered into a backward stepwise binary logistic regression model to identify independent predictors of falls. Age, gender and MMSE were directly put into multivariate logistic regression as covariates. Results are reported as odds ratios (OR) with corresponding 95% confidence intervals (CI). We used the commonly used efficacy analysis software G*power to estimate the post sample size based on the core model parameters actually analyzed in this study. The sample size of 45 cases (including 15 falls) in this study is insufficient for robust detection of risk factors through multivariate model. We have marked the results of multivariate logistic regression as “exploratory analysis,” and emphasized its instability and the need for verification in larger cohorts.

For VBM analyses, two-sample t-tests within the general linear model framework covaried for age, sex, and total intracranial volume (TIV). At the voxel level, *p* < 0.001 was used as the threshold of clump formation to define candidate clumps. FDR correction based on clumps was furtherly adopted (*p* < 0.05).

## 3 Results

### 3.1 Demographic and neuropsychological comparisons between faller and non-faller groups

The study enrolled 24 aMCI and 21 AD patients (30 non-fallers, 15 fallers). The average ages of aMCI and AD group were 64.73 ± 1.32 and 66.74 ± 1.14 years old, respectively. The average MMSE scores of aMCI and AD group were 23.9 ± 0.28 and 17.7 ± 0.90, respectively. AD Faller group included 8 patients with aMCI and 7 patients with AD. The results showed that the faller group consisted of 8 males, while the non-faller group included 16 males. The median age was 66.00 years (IQR 65.00, 68.00) for fallers and 65.00 years (IQR 62.25, 68.00) for non-fallers. Education level did not differ significantly between groups, with both medians ranging from 9 to 12 years (*p* = 0.623). Furthermore, there were no significant differences between fallers and non-fallers in the prevalence of hypertension (HBP), diabetes mellitus (DM), coronary heart disease (CHD), or hyperlipidemia (HLP) (all *p* > 0.05). In terms of physiological indicators, BMI differed significantly between groups (*p* = 0.019). Fallers had a lower mean BMI (23.20 ± 2.93 kg/m^2^) than non-fallers (25.00 ± 1.98 kg/m^2^). Moreover, a history of falls in the preceding year was reported by 20.0% of fallers compared with 6.7% of non-fallers; however, this difference was not statistically significant (*p* = 0.402). Medication burden was significantly higher in fallers [median (IQR) 3.00 (3.00, 4.00)] than in non-fallers [2.00 (1.00, 2.75); *p* < 0.001]. There were no significant differences between fallers and non-fallers in scores on the MMSE (*p* = 0.885), CVLT total score (*p* = 0.317), DST total score (*p* = 0.693), or BNT (*p* = 0.698). Notably, the TMT B-A time was significantly longer in fallers [median (IQR) 85.00 (67.00, 105.50) seconds] compared to non-fallers [58.00 (47.25, 93.00) seconds; *p* = 0.033], suggesting that poorer executive function may be associated with increased fall risk (Table 1).

**Table 1:** Demographic and neuropsychological characteristics of non-faller and faller groups.

General information and assessme	Medical history	Non-faller (*n* = 30)	Faller (*n* = 15)	*Z*/*t*/*χ*^2^ Value	*p* Value	Cohen’s *d* value
Gender male (%)		15 (50.00%)	8 (53.33%)	0.044	0.833	0.031
Age (years)		65.00 (62.25, 68.00)	66.00 (65.00, 68.00)	0.653	0.514	0.035
Education level (years)		10.00 (9.00, 12.00)	9.00 (9.00, 13.00)	0.491	0.623	0.109
Hypertension (%)	No	23 (76.67%)	10 (66.67%)	0.128	0.721	0.107
	Yes	7 (23.33%)	5 (33.33%)			
Diabetes (%)	No	27 (90.00%)	12 (80.00%)	0.216	0.642	0.139
	Yes	3 (10.00%)	3 (20.00%)			
Coronary heart disease (%)	No	28 (93.33%)	14 (93.33%)	0.000	1.000	0.000
	Yes	2 (6.67%)	1 (6.67%)			
Hyperlipidemia (%)	No	29 (96.67%)	14 (93.33%)	0.000	1.000	0.076
	Yes	1 (3.33%)	1 (6.67%)			
BMI		25.00 ± 1.98	23.20 ± 2.93	2.438	**0.019**	**0.771**
Fallen in past year (%)	No	28 (93.33%)	12 (80.00%)	0.703	0.402	0.200
	Yes	2 (6.67%)	3 (20.00%)			
Number of medications		2.00 (1.00, 2.75)	3.00 (3.00, 4.00)	3.595	**<0.001**	**1.319**
MMSE		23.00 (13.25, 25.00)	22.00 (16.50, 24.00)	0.145	0.885	0.069
CVLT total		15.00 (11.00, 21.75)	14.00 (10.00, 19.50)	1.001	0.317	0.377
DST total		9.13 ± 1.28	9.33 ± 2.09	0.398	0.693	
TMT B-TMT A		58.00 (47.25, 93.00)	85.00 (67.00, 105.50)	2.132	**0.033**	**0.625**
BNT		20.90 ± 4.30	20.40 ± 3.46	0.391	0.698	0.124

The bold values indicated *p* < 0.05 and the corresponding Cohen’s d Value.

### 3.2 Comparison of gait parameters between non-faller and faller groups

During single-task walking (STW), there were no significant differences between fallers and non-fallers in step width (*p* = 0.325) or step height (*p* = 0.586). However, significant differences were observed for stride velocity and stride length variability. Fallers walked significantly slower than non-fallers (mean stride velocity: 77.00 ± 10.35 cm/s vs. 83.67 ± 9.60 cm/s; *p* = 0.038). Stride length variability was significantly higher in fallers [median (IQR) 9.21 (5.67, 14.42)] compared to non-fallers [5.96 (4.64, 7.18); *p* = 0.003]. These findings indicated poorer gait control in fallers, characterized by reduced stride velocity and increased stride length variability. In contrast, there were no significant between-group differences in the dual-task cost (DTC) for stride velocity, either for the calculation task (DTW-Cal DTC; *p* = 0.523) or the verbal fluency task (DTW-VF DTC; *p* = 0.109) (Table 2).

**Table 2:** Comparison of gait parameters between non-faller and faller groups.

Gait parameters	Non-faller (*n* = 30)	Faller (*n* = 15)	*Z*/*t* value	*p* value	Cohen’s *d* value
Single-task					
Step width (cm)	13.00 (12.00, 13.75)	13.00 (12.00, 14.00)	0.984	0.325	0.360
Step height (cm)	12.50 (11.62, 13.50)	11.50 (10.50, 13.50)	0.545	0.586	0.097
Stride velocity (cm/s)	83.67 ± 9.60	77.00 ± 10.35	2.140	**0.038**	**0.677**
Stride length variability (%)	5.96 (4.64, 7.18)	9.21 (567, 14.42)	2.974	**0.003**	**1.268**
Dual-task					
DTW-Cal DTC (%)	22.24 (20.35, 22.86)	22.94 (19.17, 27.68)	0.638	0.523	0.400
DTW-VF DTC (%)	16.11 (10.45, 18.72)	18.56 (17.10, 21.45)	1.602	0.109	0.553

The bold values indicated *p* < 0.05 and the corresponding Cohen’s d Value.

### 3.3 Independent predictors of falls: multivariate logistic regression

**Figure 1:**
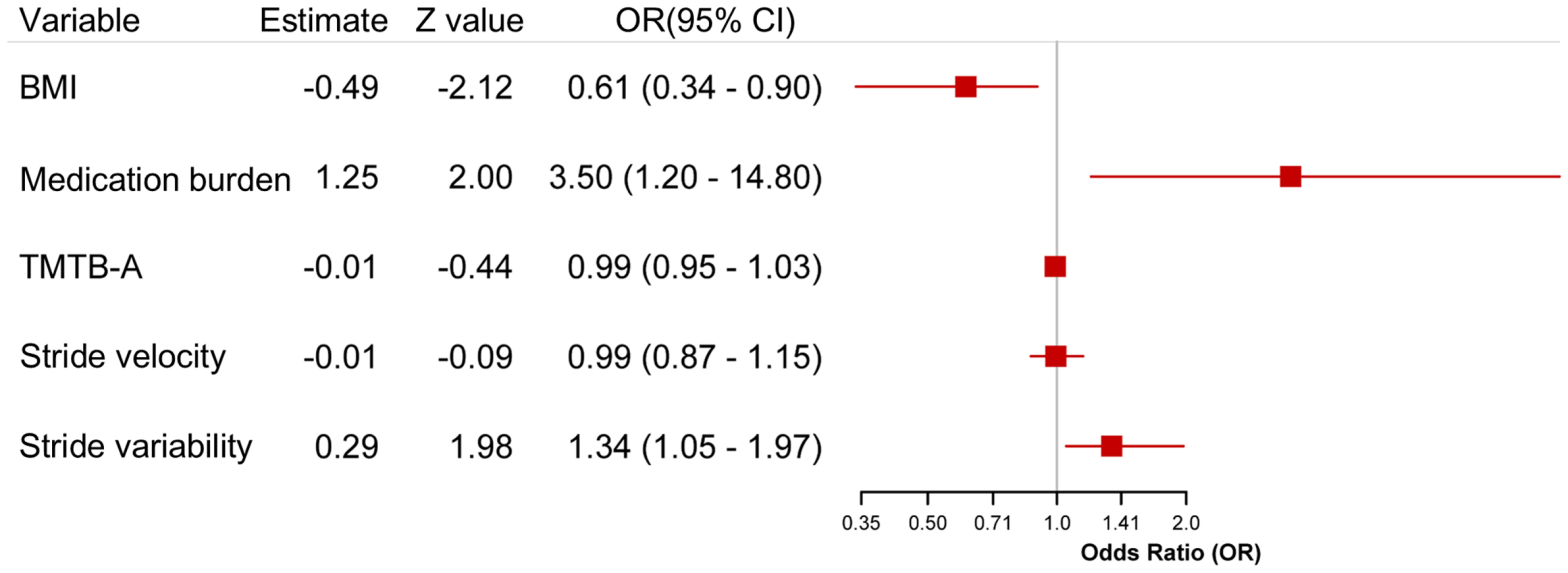
Forest plot of future fall risk predictors in AD-derived MCI and Alzheimer’s disease cohorts. Forest chart showed the independent predictors of future fall risk in patients with AD derived MCI and AD after correction of matching factors (age, gender, MMSE). Logistic regression was used in the analysis, and the independent risk factors were expressed by odds ratio (OR) and its 95% confidence interval (95% CI).

Multivariate logistic regression analysis suggested that neither TMT B-A time (OR = 0.990, 95% CI = 0.949–1.033; *p* = 0.657) nor stride velocity (OR = 0.994, 95% CI = 0.869–1.137; *p* = 0.931) was significantly associated with falls. In contrast, lower BMI (OR = 0.613, CI = 0.390–0.965; *p* = 0.034), higher medication burden (OR = 3.500, 95% CI = 1.023–11.977; *p* = 0.046), and increased stride length variability (OR = 1.341, 95% CI = 1.004–1.791; *p* = 0.047) may emerged as independent predictors of fall risk (Table 3; Figure 1). In the limited sample AD queue, the findings were are preliminary and exploratory. Since the ratio of the number of predictive variables to the number of events is not ideal, these results should be interpreted carefully, mainly for generating future assumptions.

**Table 3:** Multivariate regression analysis of independent fall-related parameters.

Potential risk factors	*B*	SE	*Z* value	OR (95%CI)	*p* value
Constant	6.582	9.156	0.517		0.472
BMI	−0.489	0.231	4.477	0.613 (0.390, 0.965)	**0.034**
Medication burden	1.253	0.628	3.985	3.500 (1.023, 11.977)	**0.046**
TMT B-A time	−0.010	0.022	0.197	0.990 (0.949, 1.033)	0.657
Stride length variability	0.293	0.148	3.937	1.341 (1.004, 1.791)	**0.047**
Stride velocity	−0.006	0.068	0.008	0.994 (0.869, 1.137)	0.931

The bold values indicated *p* < 0.05.

### 3.4 Brain structure differences between fallers and non-fallers

**Figure 2:**
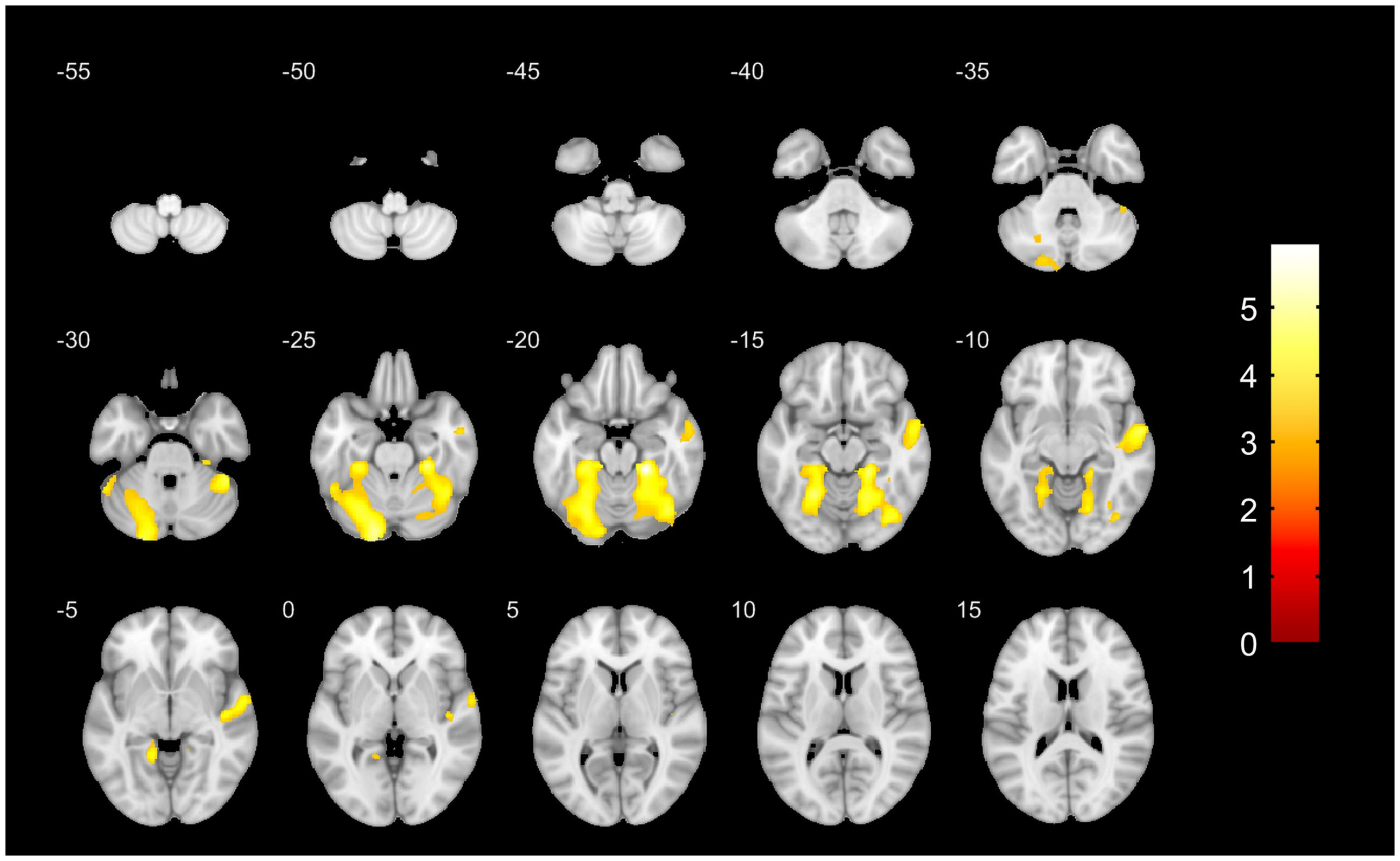
Spatial patterns of cerebral atrophy in fallers with AD-derived MCI and Alzheimer’s dementia. VBM analysis showed the yellow regions represented significantly reduced volume in faller versus no fallers and the color bar represented the reduction degree of local gray matter volume (*T* value).

Compared with non-fallers, fallers exhibited significantly reduced gray matter volume in the following regions: left cerebellar Crus I, lobule VI, and fusiform gyrus; right cerebellar lobule VI, fusiform gyrus, lobule IV–V; and right superior and middle temporal gyrus (Table 4; Figure 2). Reduced gray matter volume in the above brain region may be associated with increased risk of falls of AD patients, which need to be further validated in larger cohorts. These VBM findings were based on preliminary observations of small samples.

**Table 4:** Gary matter volume differences between non-faller and faller groups.

Anatomical region (AAL atlas)	Voxel	MNI coordinates			*T* Value
		*X*	*Y*	*Z*	
L: cerebellar crus I, lobule VI, fusiform gyrus	6,914	−16.5	−91.5	−27	5.1946
R: cerebellar lobule VI, fusiform gyrus, lobule IV–V	5,325	22.5	−34.5	−19.5	5.9371
R: superior temporal gyrus, middle temporal gyrus	1812	58.5	−4.5	−9	4.7115

AAL is automated anatomical labeling; MNI: Montreal Neurological Institute.

## 4 Discussion

Falls represent a major threat to the physical and mental health of older adults. Patients with AD exhibit a significantly higher incidence of falls than age-matched cognitively healthy controls, which is attributable to AD-specific neuropathology (3). In this prospective nested case–control study, we identified incident fallers within a cohort of individuals with aMCI and AD. Each faller was matched 1:2 with non-fallers based on sex, age (±5 years), and MMSE score (±3 points). This design helped mitigate confounding by global cognitive function and key demographics, allowing us to focus on the association of baseline domain-specific cognition, gait parameters, and gray matter volume with incident falls. BMI, stride length variability, and medication burden were found to be independent predictors of incident falls by univariate and multivariate analyses. Furthermore, VBM analysis suggested significantly reduced gray matter volume in fallers compared to non-fallers within the left cerebellar Crus I, lobule VI, and fusiform gyrus; right cerebellar lobule VI, fusiform gyrus, and lobule IV–V; and right superior and middle temporal gyrus. These findings suggest that atrophy in these specific brain regions may contribute to increased fall risk in individuals on the AD spectrum.

Extensive evidence indicates that older adults with cognitive impairment face heightened fall risk. Impairments in specific cognitive domains have been implicated as contributing factors. Casemiro et al. (17) established executive function as a critical cognitive resource for safe ambulation, with its impairment directly elevating fall risk. During adaptive walking, executive abilities enable: path planning, rapid decision-making (e.g., obstacle avoidance), working memory (e.g., environmental mapping), response inhibition (e.g., distraction suppression). Consequently, executive impairment may precipitate falls through dysregulation of these processes. In contrast, Beauchet et al. (18) associated high fall risk with memory decline in dementia-free community dwellers, while another study using the Auditory Verbal Learning Test (AVLT) linked poor memory-particularly short-delay recall-to increased fall incidence in aMCI and mild AD (9). Conversely, Chantanachai et al. (19) found no prospective association between cognitive measures and falls. Given these inconsistent findings, our study matched fallers and non-fallers on MMSE scores (±3 points), thereby controlling for global cognition to better isolate potential domain-specific associations. Univariate analysis revealed a significant association between executive dysfunction (longer TMT B-A time) and faller status. However, this association was attenuated in the multivariate model, suggesting that executive dysfunction may influence fall risk indirectly, potentially mediated through its effects on physiological factors such as gait stability, rather than exerting a direct effect. Future large-scale prospective studies should quantify domain-specific cognitive contributions to fall pathogenesis or examine interaction effects between cognitive deficits and biomechanical risk factors.

Gait impairments belong to the strongest biomechanical correlates of falls. Contemporary analysis techniques enable precise measurement of parameters such as gait velocity-a global functional indicator-and gait variability, an emerging metric for dynamic instability assessment. While univariate analyses implicated both slower stride velocity and higher stride length variability, multivariate logistic regression prompted only higher baseline stride length variability as an independent predictor of incident falls. Consistent with our results, dual-task gait variability was found to predict incident falls in community-dwelling older adults, whereas gait velocity did not (7). Besides, a cross-sectional study found that gait variability and braking force were associated with the risk of falls in AD patients (20). Gonçalves et al. (21) reported that increased stride velocity dual-task cost (DTC) predicted falls in individuals with MCI but not in those with AD dementia. However, our study showed no significant group differences in stride velocity DTC (for either calculation or verbal fluency tasks), possibly due to simpler cognitive tasks. Seavey and Walters (22) demonstrated that gait deterioration increases with dual-task complexity, observing greater impairment during serial-7 subtraction than during category fluency tasks. In our study, which employed serial-3 subtraction and animal naming tasks, the participants’ median MMSE scores (22, 23) may have provided sufficient cognitive reserve to mitigate dual-task effects. This suggests that dual-task paradigms should be calibrated to participants’ cognitive reserve levels, as differential task demands may identify distinct high-risk subgroups. Absence of cognitive-task accuracy data may also affect the stability of the results. A Systematic Review suggested no dual-task tests predicted prospective falling in people with Alzheimer’s or Parkinson’s disease and complex dual tasks seemed to be more predictive of fall risk than simpler dual tasks (23). Furthermore, Makizako et al. (24) identified impaired balance as a critical factor elevating fall risk in MCI patients, potentially exceeding the impact of gait variability. Another study in AD populations confirmed this, showing that functional reach test (FRT), single-leg stance duration and TUG test are significantly related to falls (25). Notably, our study did not address balance metrics. Future investigations should incorporate quantitative balance assessments integrated with multivariate modeling to delineate key determinants of falls within this population.

Increasing evidence supports that lower BMI increases the risk of AD onset (26), but the association between BMI and fall risk in AD patients has not been investigated. Our findings suggested lower BMI maybe an independent predictor of falls of AD patients. Lower BMI has been considered to be associated with physical frailty, a geriatric syndrome characterized by declined physiologic reserve and heightened vulnerability to adverse outcomes-and muscle loss, which are also recognized correlates of falls in AD patients (27, 28). Implement nutritional interventions to optimize the BMI level may be prevent from fall of AD patients.

Patients with cognitive impairment, particularly moderate-to-severe AD, frequently exhibit neuropsychiatric symptoms and sleep disturbances necessitating polypharmacy. While pharmacotherapy offers benefits, polypharmacy increases the risk of adverse events, including falls. Medications particularly associated with fall risk include antidepressants, antipsychotics, and sedative-hypnotics. This study quantified the total medication burden for AD-specific cognitive treatments (ChEIs, NMDA receptor antagonists), antidepressants, antipsychotics, and benzodiazepines. The results suggested total medication count might independently predicted incident falls, aligning with findings from Cox et al. (29). In previous literature, ChEIs may improve gait and balance control by enhancing attention and executive function, potentially reducing fall risk (30). However, case reports also link ChEIs (e.g., donepezil) to syncope (31) and NMDA receptor antagonists (e.g., memantine) to abnormal cardiac rate (32). These findings underscore the necessity to carefully weigh the potential benefits of pharmacotherapy against the risk of serious adverse events (including fall risk), when considering treatment efficacy.

Accumulating neuroimaging evidence confirms brain-physical function linkages, yet the neuropathological mechanisms specifically underlying falls remain poorly elucidated. To our knowledge, this study provides the first evidence linking specific patterns of gray matter volume reduction to falls within the AD spectrum. Whole-brain VBM analysis showed compared to matched non-fallers, gray matter volumes within the left cerebellar Crus I, lobule VI, and fusiform gyrus; the right cerebellar lobule VI, fusiform gyrus, and lobule IV–V; and the right superior and middle temporal gyrus were significantly reduced in fallers. The results of VBM analysis suggested that reduced gray matter volume in specific brain region might be associated with increased risk of falls of AD patients. In agreement with our results, a resting state functional MRI found that Cerebello-cortical functional connectivity might regulate impaired reactive balance control in older adults with mild cognitive impairment, which may be associated with increased fall risk (33). A small sample case–control study collecting 14 elderly patients with falls and 20 controls found elderly patients with falls exhibited lower cerebellar volumes in the posterior cerebellum, lobules V, VI, VIIB, VIIIA, VIIIB, and Crus II, compared to control group, suggesting cerebellar atrophy contributes to the pathophysiology of fall risk in elderly fallers (34). It was reported that cerebellar cortical volume reduced persisting from early to late clinical stages of AD (35). Notably, atrophy in anterior (lobules I-V) and posterior (lobule VI) cerebellar lobes has been observed in the aMCI stage, with later involvement of the hemispheric portions of the posterior lobe (VI) and Crus I in AD (36). Neuropathological studies confirm the presence of AD pathology in the cerebellum, including Aβ deposition (though typically less dense than in the medial temporal cortex) (37) and p-Tau accumulation (38). Functional neuroimaging and clinical studies indicate that the cerebellum participates in diverse cognitive functions, including working memory, learning, attention, and executive control (39, 40). Specifically, Crus I and Crus II appear to modulate executive control and visuospatial memory (41). The findings of VBM were preliminary and observational based on the small sample size. The specific patterns of gray matter volume reduction may contribute to the increased fall risk in patients with AD, which need to be further validated in larger cohorts.

In generally, the preliminary findings of this study indicate that BMI, stride length variability, and medication burden may be associated with fall risk and reduced gray matter volume in specific brain region (cerebellum and the right superior and middle temporal gyrus) may be a potential neuroanatomical basis for increased risk of falls in AD patients. Several limitations should be acknowledged: (1) the small sample size limits the robustness of the predictive model and generalizability; findings require validation in larger, independent cohorts; (2) the exclusive focus on gray matter volume warrants future integration with fMRI and more detailed behavioral phenotyping to elucidate mechanisms; (3) the contributions of key subcortical nuclei involved in gait regulation are not examined and represent a critical target for future research.

## Data Availability

The raw data supporting the conclusions of this article will be made available by the authors, without undue reservation.

## References

[1] Sherrington C Fairhall N Wallbank G Tiedemann A Michaleff ZA Howard K . Exercise for preventing falls in older people living in the community: an abridged Cochrane systematic review. Br J Sports Med. (2020) 54:885–91. doi: 10.1136/bjsports-2019-101512, 31792067

[2] Hausdorff JM Rios DA Edelberg HK. Gait variability and fall risk in community-living older adults: a 1-year prospective study. Arch Phys Med Rehabil. (2001) 82:1050–6. doi: 10.1053/apmr.2001.24893 11494184

[3] Dyer AH Lawlor B Kennelly SP NILVAD Study Group. Gait speed, cognition and falls in people living with mild-to-moderate Alzheimer disease: data from NILVAD. BMC Geriatr. (2020) 20:117. doi: 10.1186/s12877-020-01531-w, 32228468 PMC7106668

[4] Allan LM Ballard CG Rowan EN Kenny RA. Incidence and prediction of falls in dementia: a prospective study in older people. PLoS One. (2009) 4:e5521. doi: 10.1371/journal.pone.0005521, 19436724 PMC2677107

[5] de Oliveira Silva F Ferreira JV Plácido J Chagas D Praxedes J Guimarães C . Stages of mild cognitive impairment and Alzheimer’s disease can be differentiated by declines in timed up and go test: a systematic review and meta-analysis. Arch Gerontol Geriatr. (2019) 85:103941. doi: 10.1016/j.archger.2019.103941, 31476630

[6] Lee HS Ko M Park SW Braden H. Concurrent validity of the Groningen meander walking and timed up and go tests in older adults with dementia. Physiother Theory Pract. (2020) 36:1432–7. doi: 10.1080/09593985.2019.1579285, 30739570

[7] Zhang C Dong X Ding M Chen X Shan X Ouyang H . Executive control, alerting, updating, and falls in cognitively healthy older adults. Gerontology. (2020) 66:494–505. doi: 10.1159/000509288, 32841943

[8] Sheridan PL Hausdorff JM. The role of higher-level cognitive function in gait: executive dysfunction contributes to fall risk in Alzheimer’s disease. Dement Geriatr Cogn Disord. (2007) 24:125–37. doi: 10.1159/000105126, 17622760 PMC3163262

[9] Huang S Zhou X Liu Y Luo J Lv Z Shang P . High fall risk associated with memory deficit and brain lobes atrophy among elderly with amnestic mild cognitive impairment and mild Alzheimer’s disease. Front Neurosci. (2022) 16:896437. doi: 10.3389/fnins.2022.896437, 35757554 PMC9213689

[10] Keleman AA Nicosia J Bollinger RM Wisch JK Hassenstab J Morris JC . Precipitating mechanisms of falls in preclinical Alzheimer’s disease. J Alzheimers Dis Rep. (2023) 7:739–50. doi: 10.3233/ADR-230002, 37483329 PMC10357117

[11] Horikawa E Matsui T Arai H Seki T Iwasaki K Sasaki H. Risk of falls in Alzheimer’s disease: a prospective study. Intern Med. (2005) 44:717–21. doi: 10.2169/internalmedicine.44.717, 16093593

[12] Keleman A Wisch JK Bollinger RM Grant EA Benzinger TL Morris JC . Falls associate with neurodegenerative changes in ATN framework of Alzheimer’s disease. J Alzheimer's Dis. (2020) 77:745–52. doi: 10.3233/jad-200192, 32741815 PMC7580016

[13] Allali G Annweiler C Predovan D Bherer L Beauchet O. Brain volume changes in gait control in patients with mild cognitive impairment compared to cognitively healthy individuals; GAIT study results. Exp Gerontol. (2016) 76:72–9. doi: 10.1016/j.exger.2015.12.007, 26705916

[14] Albert MS DeKosky ST Dickson D Dubois B Feldman HH Fox NC . The diagnosis of mild cognitive impairment due to Alzheimer’s disease: recommendations from the National Institute on Aging-Alzheimer’s association workgroups on diagnostic guidelines for Alzheimer’s disease. Alzheimers Dement. (2011) 7:270–9. doi: 10.1016/j.jalz.2011.03.008 21514249 PMC3312027

[15] Hyman BT Phelps CH Beach TG Bigio EH Cairns NJ Carrillo MC . National Institute on Aging-Alzheimer’s association guidelines for the neuropathologic assessment of Alzheimer’s disease. Alzheimers Dement. (2012) 8:1–13. doi: 10.1016/j.jalz.2011.10.007 22265587 PMC3266529

[16] Llinàs-Reglà J Vilalta-Franch J López-Pousa S Calvó-Perxas L Torrents Rodas D Garre-Olmo J. The trail making test. Assessment. (2017) 24:183–96. doi: 10.1177/1073191115602552, 26318386

[17] Casemiro FG Carvalho LPND Matiello FDB Resende MC Rodrigues RAP. Influence of frailty and cognitive decline on dual task performance in older adults: an analytical cross-sectional study. Rev Lat Am Enfermagem. (2025) 33:e4485. doi: 10.1590/1518-8345.7159.4485 39969040 PMC11835002

[18] Beauchet O Allali G Montero-Odasso M Sejdić E Fantino B Annweiler C. Motor phenotype of decline in cognitive performance among community-dwellers without dementia: population-based study and meta-analysis. PLoS One. (2014) 9:e99318. doi: 10.1371/journal.pone.0099318, 24911155 PMC4049832

[19] Chantanachai T Taylor ME Lord SR Menant J Delbaere K Sachdev PS . Risk factors for falls in community-dwelling older people with mild cognitive impairment: a prospective one-year study. PeerJ. (2022) 10:e13484. doi: 10.7717/peerj.13484, 35663527 PMC9161814

[20] Cheng Q Wu M Wu Y Hu Y Kwapong WR Shi X . Weaker braking force, a new marker of worse gait stability in Alzheimer disease. Front Aging Neurosci. (2020) 12:554168. doi: 10.3389/fnagi.2020.554168, 33024432 PMC7516124

[21] Gonçalves J Ansai JH Masse FAA Vale FAC de Medeiros Takahashi AC de Andrade LP. Dual-task as a predictor of falls in older people with mild cognitive impairment and mild Alzheimer’s disease: a prospective cohort study. Braz J Phys Ther. (2018) 22:417–23. doi: 10.1016/j.bjpt.2018.03.011 29636306 PMC6158075

[22] Seavey CV Walters BH. Motor dual-task deficits and their associations with executive function in older adults with cognitive impairments. J Mot Behav. (2025) 57:502–18. doi: 10.1080/00222895.2025.2514483, 40497469

[23] Peters J Lauinger A Mayr M Ginell K Abou L. Dual-task assessments for predicting future falls in neurologic conditions: a systematic review. Am J Phys Med Rehabil. (2024) 103:554–60. doi: 10.1097/PHM.0000000000002452, 38466165

[24] Makizako H Shimada H Doi T Park H Yoshida D Uemura K . Poor balance and lower gray matter volume predict falls in older adults with mild cognitive impairment. BMC Neurol. (2013) 5:102. doi: 10.1186/1471-2377-13-102 PMC3750260 23915144

[25] Oki M Matsumoto M Yoshikawa Y Fukushima M Nagasawa A Takakura T . Risk factors for falls in patients with Alzheimer disease: a retrospective study of balance, cognition, and visuospatial ability. Dement Geriatr Cogn Dis Extra. (2021) 11:58–63. doi: 10.1159/000514285, 33976693 PMC8077477

[26] Lee EH Yoo H Kim YJ Cheon BK Ryu S Chang Y . Different associations between body mass index and Alzheimer’s markers depending on metabolic health. Alzheimer's Res Ther. (2024) 16:194. doi: 10.1186/s13195-024-01563-z, 39210402 PMC11363444

[27] Güner Oytun M Topuz S Baş AO Çöteli S Kahyaoğlu Z Boğa İ . Relationships of fall risk with frailty, sarcopenia, and balance disturbances in mild-to-moderate Alzheimer’s disease. J Clin Neurol. (2023) 19:251–9. doi: 10.3988/jcn.2022.0219, 36647232 PMC10169927

[28] de Souza LF Canever JB Moreira B d S Danielewicz AL de Avelar NCP. Association between fear of falling and frailty in community-dwelling older adults: a systematic review. Clin Interv Aging. (2022) 17:129–40. doi: 10.2147/CIA.S328423 35173427 PMC8843349

[29] Cox CA van Jaarsveld HJ Houterman S van der Stegen JCGH Wasylewicz ATM Grouls RJE . Psychotropic drug prescription and the risk of falls in nursing home residents. J Am Med Dir Assoc. (2016) 17:1089–93. doi: 10.1016/j.jamda.2016.07.004 27650670

[30] Montero-Odasso M Speechley M Chertkow H Sarquis-Adamson Y Wells J Borrie M . Donepezil for gait and falls in mild cognitive impairment: a randomized controlled trial. Eur J Neurol. (2019) 26:651–9. doi: 10.1111/ene.13872, 30565793

[31] Ahuja M Siddhpuria S Karimi A Lewis K Wong E Lee J . Cholinesterase inhibitors and falls, syncope and injuries in patients with cognitive impairment: a systematic review and meta-analysis. Age Ageing. (2023) 52:afad205. doi: 10.1093/ageing/afad205, 37993407

[32] Aksoyalp ZŞ Nemutlu-Samur D. Comparative post-marketing surveillance of memantine and cholinesterase inhibitors: cardiovascular adverse events with a focus on sex differences using the FDA adverse event reporting system database. Int J Geriatr Psychiatry. (2024) 39:e70018. doi: 10.1002/gps.70018, 39562528

[33] Kannan L Bhatt T Ajilore O. Cerebello-cortical functional connectivity may regulate reactive balance control in older adults with mild cognitive impairment. Front Neurol. (2023) 14:1041434. doi: 10.3389/fneur.2023.1041434, 37139074 PMC10149739

[34] Droby A El Mendili MM Giladi N Hausdorff JM Maidan I Mirelman A. Gait and cognitive abnormalities are associated with regional cerebellar atrophy in elderly fallers—a pilot study. Gait Posture. (2021) 90:99–105. doi: 10.1016/j.gaitpost.2021.08.012 34428633

[35] Tabatabaei-Jafari H Walsh E Shaw ME Cherbuin N. Alzheimer’s Disease Neuroimaging Initiative (ADNI). The cerebellum shrinks faster than normal ageing in Alzheimer’s disease but not in mild cognitive impairment. Hum Brain Mapp. (2017) 38:3141–50. doi: 10.1002/hbm.23580, 28321950 PMC5426955

[36] Samstag CL Chapman NH Gibbons LE Geller J Loeb N Dharap S . Neuropathological correlates of vulnerability and resilience in the cerebellum in Alzheimer’s disease. Alzheimers Dement. (2025) 21:e14428. doi: 10.1002/alz.14428, 39713867 PMC11848203

[37] Lopez G Magaki SD Williams CK Paganini-Hill A Vinters HV. Characterization of cerebellar amyloid-β deposits in Alzheimer disease. J Neuropathol Exp Neurol. (2024) 83:72–8. doi: 10.1093/jnen/nlad107, 38114098 PMC10799296

[38] Sepulveda-Falla D Matschke J Bernreuther C Hagel C Puig B Villegas A . Deposition of hyperphosphorylated tau in cerebellum of PS1 E280A Alzheimer’s disease. Brain Pathol. (2011) 21:452–63. doi: 10.1111/j.1750-3639.2010.00469.x, 21159009 PMC8094246

[39] Küper M Kaschani P Thürling M Stefanescu MR Burciu RG Göricke S . Cerebellar fMRI activation increases with increasing working memory demands. Cerebellum. (2016) 15:322–35. doi: 10.1007/s12311-015-0703-7 26202670

[40] Tan JB Orlando IF Whyte C Bryant AG Munn BR Baracchini G . Cerebellar and subcortical contributions to working memory manipulation. Commun Biol. (2025) 8:1028. doi: 10.1038/s42003-025-08467-0, 40634487 PMC12241541

[41] Kim LH Heck DH Sillitoe RV. Cerebellar functions beyond movement and learning. Annu Rev Neurosci. (2024) 47:145–66. doi: 10.1146/annurev-neuro-100423-104943, 38663092 PMC13371864

