# Supplementary figures and images for: Clinical study on gray matter volume reduction, gait disorders, and fall risk in patients with Alzheimer’s disease
Source: Front Neurol. 2026 Feb 19;17:1737591. doi: 10.3389/fneur.2026.1737591 (PMC12960512; doi:10.3389/fneur.2026.1737591)

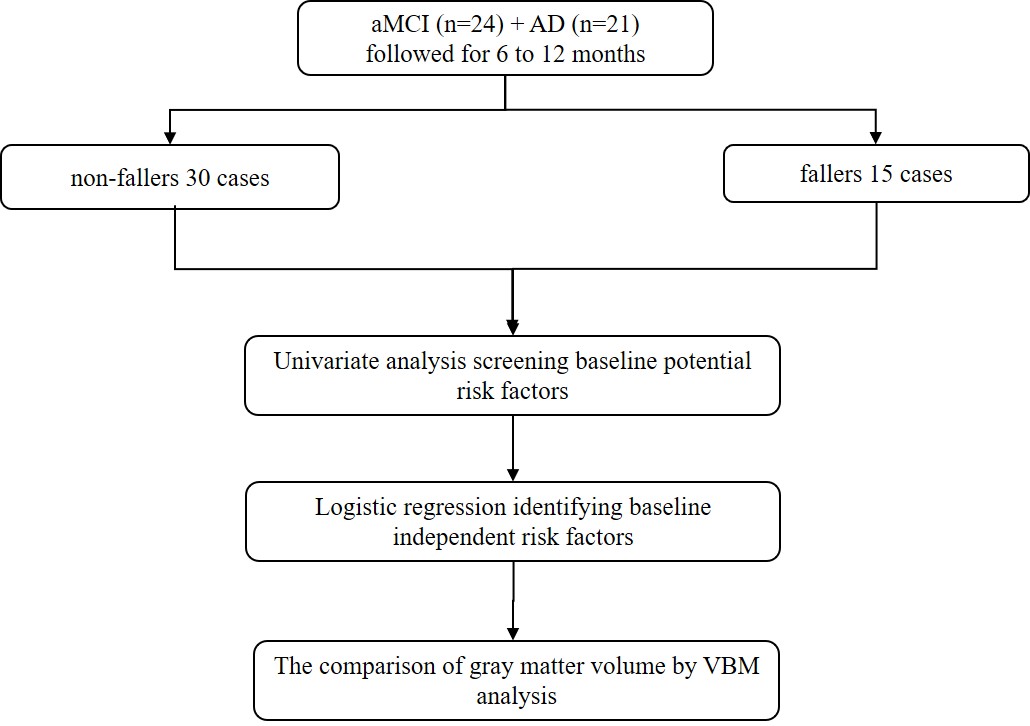

Supplement: Supplementary file 1 [file Image_1.JPEG]
